# A Novel Nomogram for the Prediction and Evaluation of Prognosis in Patients with Early-onset Kidney Cancer: a Population-based Study

**DOI:** 10.7150/jca.104569

**Published:** 2025-01-13

**Authors:** Dingyang Lv, Qiwei Wang, Ke Sun, Jinshuai Li, Huiyu Zhou, Jie Wen, Weibing Shuang

**Affiliations:** 1 Department of Urology, First Hospital of Shanxi Medical University, Taiyuan, Shanxi Province, China.; 2 First Clinical Medical College of Shanxi Medical University, Taiyuan, Shanxi Province, China.; 3 Department of Pathology, First Hospital of Shanxi Medical University, Taiyuan, Shanxi Province, China.

**Keywords:** Early-onset kidney cancer, Nomogram, Overall survival, prognosis, SEER

## Abstract

**Background:** Early-onset kidney cancer (EOKC) is often associated with genetic factors and a high risk of metastasis. However, there is a lack of accurate prediction models for the prognosis of EOKC. The aim of this study is to establish an effective nomogram for predicting and evaluating the prognosis of patients with EOKC.

**Methods:** The patients with EOKC were selected from the latest SEER database during 2004-2015. Patients between 2004 and 2014 were randomly divided into a training cohort and a validation cohort at a ratio of 7:3, and patients in 2015 were used for temporal external validation. Additionally, we included patients from First Hospital of Shanxi Medical University between 2013 and 2021 for spatial external validation. The performance of the nomogram was assessed using the concordance index (C-index), receiver operating characteristic (ROC) curves, calibration curves, and decision curve analysis (DCA). Patients were stratified based on the nomogram, and Kaplan-Meier (KM) curves were plotted to compare the survival probability of patients.

**Results:** In the temporal and spatial external validation cohort, the C-index of the nomogram for OS was 0.872 and 0.875, respectively, and the C-index of the nomogram for CSS were 0.872 and 0.851, respectively. In the temporal external validation cohort, the 1-year, 3-year and 5-year AUC of the nomogram for OS were 0.906, 0.899 and 0.876, respectively. In addition, the AUC showed that the nomogram had also high predictive ability for CSS. The calibration curves and DCA also indicated that the nomogram had a strong clinical utility. The KM curve revealed that patients in the low-risk group had a better prognosis than those in the high-risk group.

**Conclusion:** Our study developed a novel high-performance nomogram for assessing the prognosis of patients with EOKC, and it has great potential for clinicians to assess patient prognosis and formulate effective intervention and follow-up strategies.

## Introduction

Renal cell carcinoma (RCC) is one of the most prevalent malignancies in the world, representing around 3~5% of all cancers 1, 2. With the development of advanced imaging techniques, RCC can be detected and diagnosed earlier and more accurately, and its incidence is increasing annually. In 2020, there were an estimated 431,288 new cases of RCC globally 3. RCC is the most prevalent solid lesion of the kidney and accounts for approximately 85% of all kidney cancer cases 4, 5. There are different histologic subtypes of RCC with specific histopathological and genetic characteristics 6. The most prevalent subtype among them is clear cell renal cell carcinoma. There is a 1.5-2.0:1 predominance in men over women 7. An epidemiological survey in the United States found that the incidence of RCC in young people (25-49 years old) increased significantly from 1995 to 2014, with an average annual growth rate of 2.95-6.23%, surpassing that observed in the middle-aged and elderly 8. In 2014, Shuch et al. first defined early-onset kidney cancer (EOKC) as RCC diagnosed before the age of 47 (46 years or younger) 9, which may be associated with genetic factors and a higher risk of metastasis 10, 11. Truong et al.'s research reported that approximately 18% of patients with EOKC harbored a germline P/LP variant, half of which are associated with hereditary RCC syndromes, and patients with EOKC should undergo comprehensive assessment of personal and family history to guide appropriate genetic testing 12.

Accurate prediction of the prognosis of RCC is of crucial significance for clinical diagnosis and treatment. The traditional TNM staging system is the primary tool for most clinicians to evaluate the prognosis and develop diagnostic and treatment strategies for patients. However, this staging system does not include clinicopathological factors potentially influencing the prognosis of RCC, such as age, sex, basic diseases, surgical methods, histologic subtypes, grades, etc 13, 14. To overcome this limitation, Wang et al. established a nomogram to assess the prognosis of elderly patients with early RCC based on the Surveillance Epidemiology and End Results (SEER) database and validated its performance externally 15. Huang et al. established and verified a nomogram for predicting cancer-specific survival in patients with metastatic clear cell RCC 16. Leibovich et al. developed specific prognostic models for oncologic outcomes in clear cell renal cell carcinoma (ccRCC), papillary renal cell carcinoma (pRCC), and chromophobe renal cell carcinoma (chRCC), providing vital prognostic predictive information 17. These studies demonstrate the importance of clinicopathological factors as prognostic factors in predicting the prognosis and formulating treatment and follow-up strategies.

Nevertheless, there are few studies on the prognostic nomogram for EOKC, with only one reported study 18. Therefore, it is necessary to further identify the independent prognostic factors of EOKC and establish a more effective prediction model for its clinical prognosis, providing guidance for clinical diagnosis and treatment decisions. Based on the SEER database, this study divided the data from 2004 to 2014 into a training cohort and a validation cohort, and the data from 2015 was used for external validation. Additionally, we included patients from First Hospital of Shanxi Medical University for spatial external validation. The training cohort was used for Cox regression analysis to construct a prognostic nomogram, then the prediction efficiency of the model was evaluated by the receiver operating characteristic (ROC) and other methods. The novel nomogram is expected to optimize individualized treatment decisions for patients with EOKC.

## Patients and Methods

### Data source and data extraction

**Figure 1:**
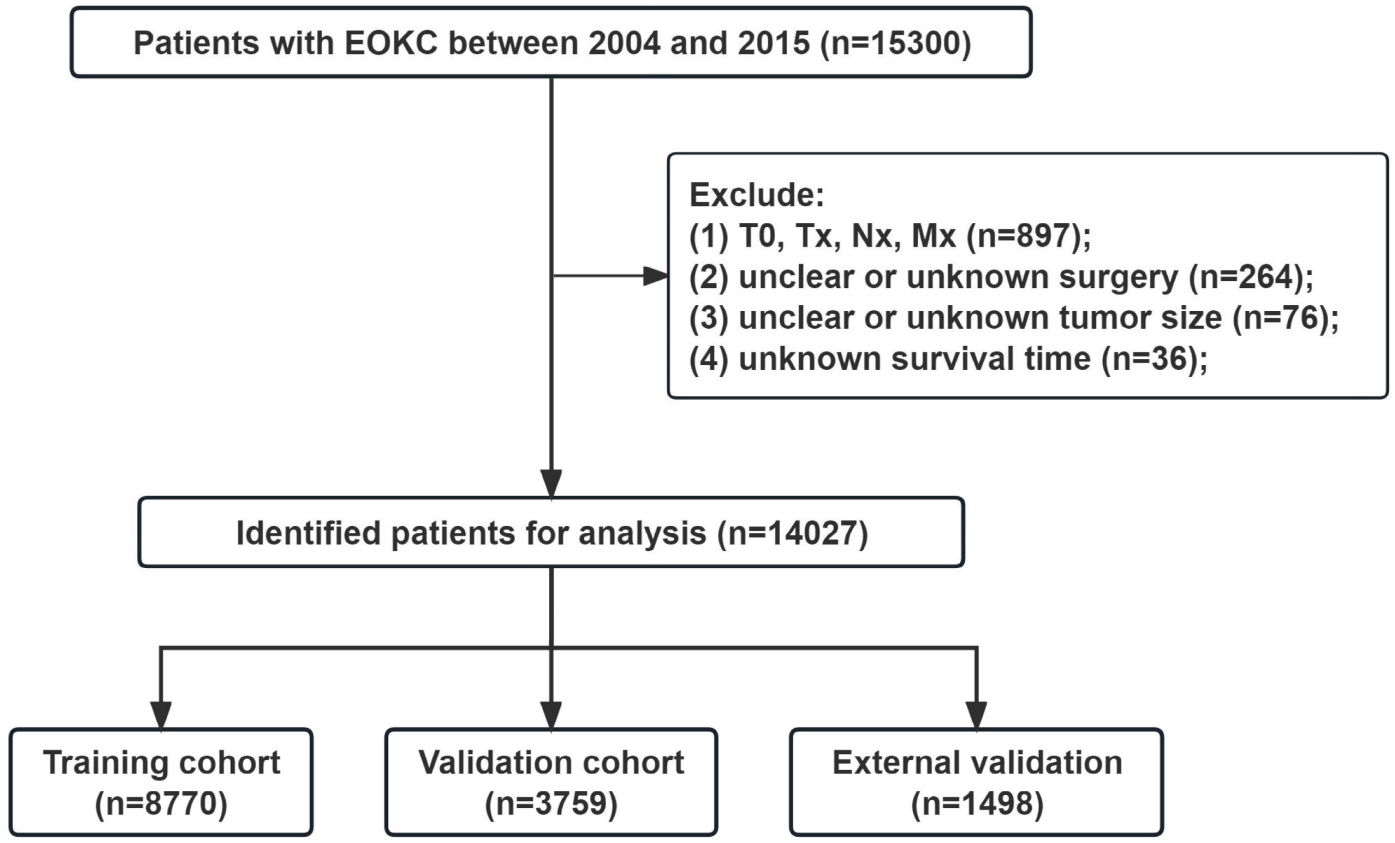
The flowchart of the EOKC patients with training and validation cohorts.

Data on young patients (aged ≤ 46 years) diagnosed with RCC from 2004 to 2015 were retrieved from the Incidence-SEER Research Data, 17 Registries, Nov 2022 Sub (2000-2020) using SEER*Stat 8.4.2. The demographic information, tumor characteristics and survival status of patients are publicly available through the SEER database. A total of 14,027 patients were included in the SEER database. Additionally, we included 176 patients from First Hospital of Shanxi Medical University between 2013 and 2021 for spatial external validation. Inclusion criteria: (1) Aged ≤ 46 years; (2) Malignant pathological diagnosis; (3) Pathological diagnosis is RCC: including RCC (8010, 8312), clear cell RCC (8310), papillary RCC (8050, 8260), chromophobe RCC (8270,8317), collecting duct carcinoma (8319), cyst-associated RCC (8316), sarcomatoid RCC (8318), medullary RCC (8510), and mixed RCC (8255, 8323); (4) unilateral tumors. Exclusion criteria: (1) T stage=T0, Tx, N stage=Nx, and M stage=Mx; (2) Unclear or unknown surgery type; (3) Unclear or unknown tumor size; (4) Unknown survival time. The flowchart for selecting patients is shown in Fig. 1. The study was approved by the ethics committee of First Hospital of Shanxi Medical University (2018K006). Informed consent was waived due to the retrospective nature of the study.

### Variable selection

Demographic and clinical data were collected, including age at diagnosis, sex, race, marital status, histological type, grade, TNM stage, AJCC stage, tumor size, radiotherapy, chemotherapy, type of surgery, survival status, and survival time. Race was categorized into black, white, and other. Marital status at diagnosis was divided into married and other (single, widowed, divorced, or separated). The pathological grade was divided into well differentiated (Grade I), moderately differentiated (Grade II), poorly differentiated (Grade III), and undifferentiated (Grade IV). The surgical methods were divided into four groups: non-surgical group, local tumor excision (photodynamic therapy, cryosurgery, thermal ablation, laser excision, et al), partial nephrectomy (PN), and radical nephrectomy (RN).

### Statistical analysis

We randomly divided 12,529 patients between 2004 and 2014 into a training cohort (n = 8770) and a validation cohort (n = 3759) at a ratio of 7:3. Patients from 2015 (n = 1498) were used for temporal external validation. Independent sample t-test and chi-square test were used to compare the clinicopathological variables between the training and validation cohorts. The Cox proportional hazards model was used for univariable and multivariable analysis to identify independent prognostic factors for EOKC. The hazard ratio (HR) and 95% confidence interval (CI) were calculated. Two new nomogram models were established using selected independent prognostic factors to estimate overall survival (OS) and cancer-specific survival (CSS) rates of patients with EOKC at 1 year, 3 years and 5 years.

The area under the ROC curve (AUC) and the Concordance Index (C-index) were calculated to evaluate the discriminative and predictive accuracy of the nomogram. The calibration curves (1000 bootstrap resamples) were constructed to validate the predictive efficiency of the nomogram. The clinical diagnostic significance of the nomogram was evaluated using Decision curve analysis (DCA), which is a new algorithm to assess the clinical utility by estimating the net benefit under each risk threshold 19. Patients were divided into low-risk and high-risk groups according to the nomogram. Kaplan-Meier curve and log-rank test were used to compare the survival rates of patients between the two groups.

All statistical analyses were conducted using R version 4.3.1. The packages, including the “rms”, “survival”, “survminer”, “survivalROC”, and “dcurves” were used to construct and validate the nomogram, formulate the ROC and calibration curves, establish DCA, draw KM curves, and perform log-rank tests. When the P value was less than 0.05, the difference was considered statistically significant (two-sided).

## Results

### Basic characteristics of patients

According to the inclusion and exclusion criteria, we included 14,027 patients with EOKC from the SEER database between 2004 and 2015. Patients from 2004 to 2014 (n=12,529) were used for the establishment and internal validation of predictive models. The mean age of the patients was 39.5±6.11 years, and there were 7,728 (61.7%) male and 4,801 (38.3%) female patients. The majority of patients were white (9,960, 79.5%), followed by black (1569, 12.5%) and other (1000, 8.0%). Among these patients, 7,114 (56.8%) were married and 5,415 (43.2%) were unmarried. The histologic subtypes of RCC included clear cell (7241, 57.8%), RCC (2422, 19.3%), papillary (1167, 9.3%), chromophobe (1007, 8.0%), and other (692, 5.5%). According to the grading system, there were 7356 (58.7%) patients with Grade I/II, 3076 (24.1%) with Grade III/IV, and 2097 (16.7%) with unknown grade. According to the AJCC staging system, there were 10,827 (86.4%) patients with T1/T2, 11,947 (95.4%) patients with N0, 11,722 (93.6%) patients with M0, and 9,050 (72.2%) patients with AJCC stage I. The mean tumor size was 5.05±4.03 cm. In addition, 278 (2.2%), 4777 (38.1%), and 6939 (55.4%) patients underwent local tumor excision, partial nephrectomy (PN), and radical nephrectomy (RN), respectively, with a small proportion of patients treated with radiotherapy (2.4%) and chemotherapy (5.2%). Patients from 2015 (n = 1498) were used for temporal external validation. The clinicopathological characteristics of EOKC patients in the training and two validation cohorts are shown in Table 1. The P-values are the results of statistical analysis comparing the training set with the two validation queues, respectively. There were no significant differences in clinicopathologic characteristics between the training cohort and two validation cohorts.

### Univariate and multivariate cox regression analysis

In the training cohort, univariate regression analyses identified 14 significant risk factors, including age, sex, race, marital status, histologic subtype, grade, TNM stage, AJCC stage, tumor size, radiotherapy, chemotherapy, and surgery type. Next, we selected these factors to establish a multivariate Cox model to determine independent prognostic factors. The results showed that age, sex, race, marital status, histologic subtype, grade, TNM stage, AJCC stage, tumor size, radiotherapy, and surgery type were independent prognostic factors for OS in patients (Table 2). At the same time, univariate and multivariate analysis suggested that sex, race, histologic subtype, grade, TNM stage, AJCC stage, tumor size, radiotherapy, and surgery type were independent prognostic factors for CSS in patients (Table 3).

### Nomogram construction for the prediction of 1-, 3-, and 5-year OS and CSS

**Figure 2:**
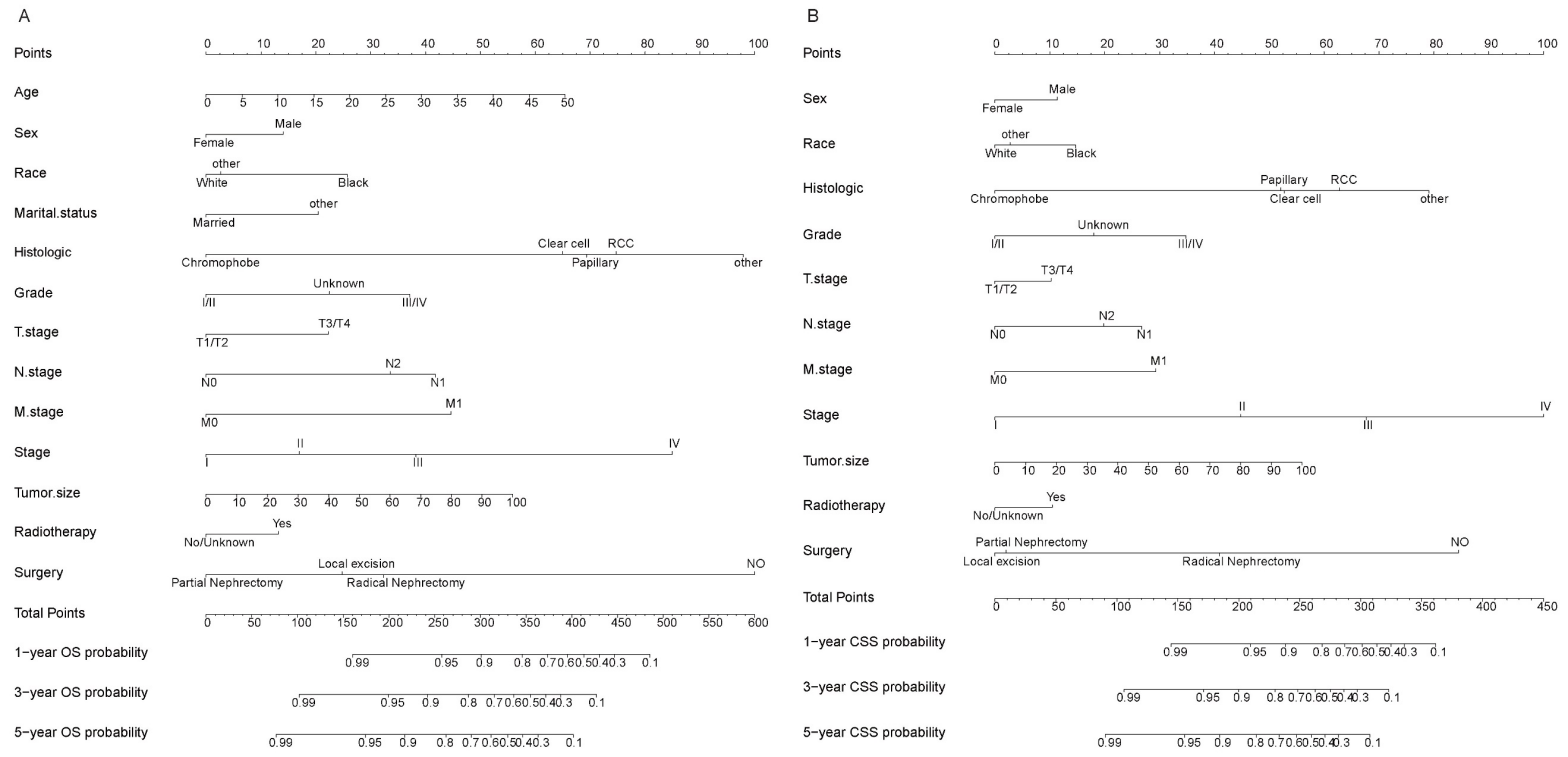
Nomograms for the prognosis in patients with EOKC (Age, years; Tumor size, mm). (A) Nomogram for prediction of 1-, 3-, and 5-year OS. (B) Nomogram for prediction of 1-, 3-, and 5-year CSS.

A nomogram model was constructed using the selected independent prognostic factors and the corresponding score for each parameter was listed (Fig. 2A). The nomogram could predict the 1-, 3- and 5-year OS. The line length of each variable in the nomogram represents its contribution to OS. The longer line length indicates the larger contribution. As shown in the nomogram, the surgery type was the strongest prognostic factor for OS, followed by AJCC stage and histologic subtype. Meanwhile, we constructed a nomogram to predict CSS in patients (Fig. 2B).

### Validation of the nomogram

**Figure 3:**
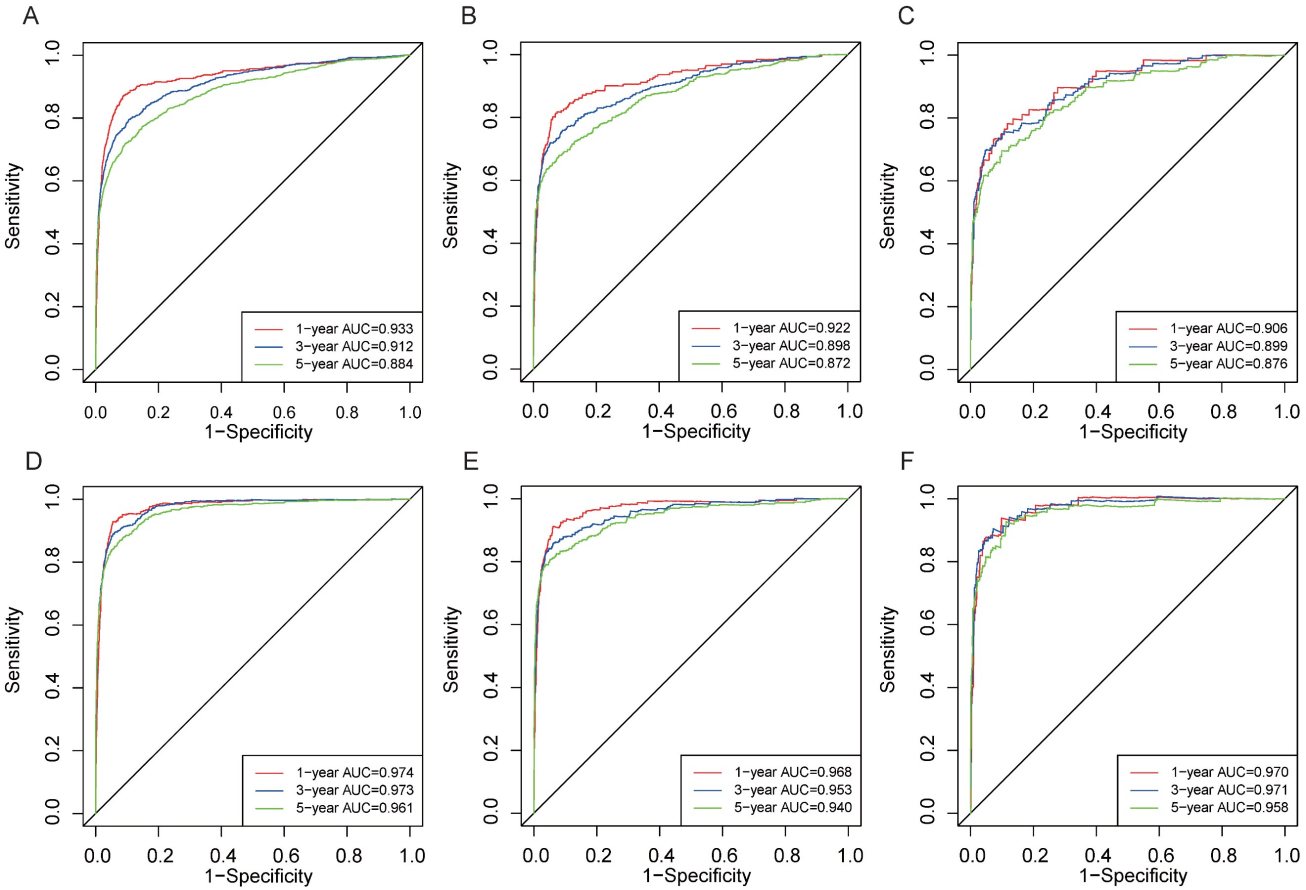
The ROC for 1-, 3- and 5-year prognosis. (A) The ROC of nomogram for OS in training cohort. (B) The ROC of nomogram for OS in validation cohort. (C) The ROC of nomogram for OS in external validation cohort. (D) The ROC of nomogram for CSS in the training cohort. (E) The ROC of nomogram for CSS validation cohort. (F) The ROC of nomogram for CSS external validation cohort.

**Figure 4:**
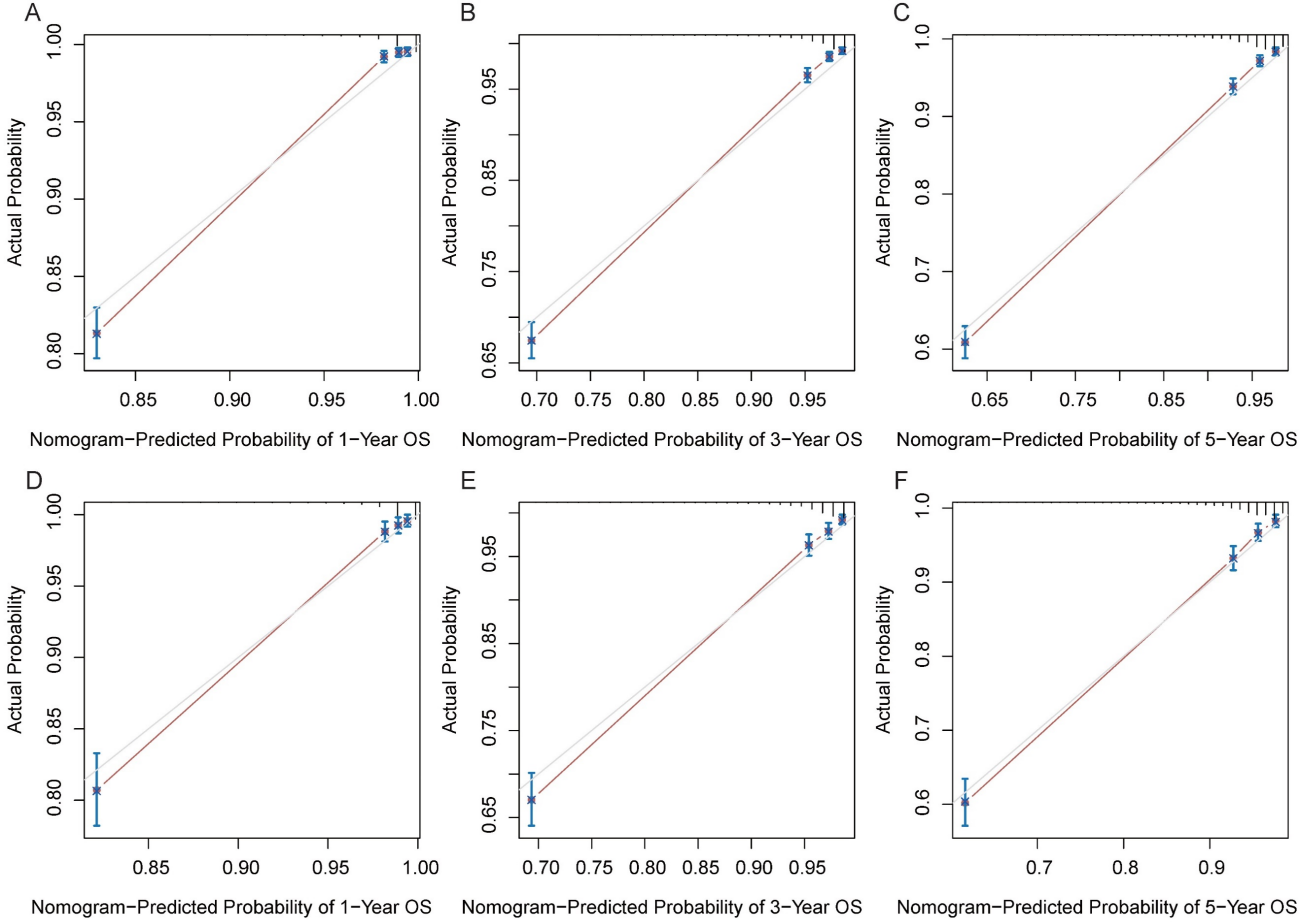
Calibration curves of the nomogram. (A) 1-year OS in the training cohort. (B) 3-year OS in the training cohort. (C) 5-year OS in the training cohort. (D) 1-year OS in the validation cohort. (E) 3-year OS in the validation cohort. (F) 5-year OS in the validation cohort.

**Figure 5:**
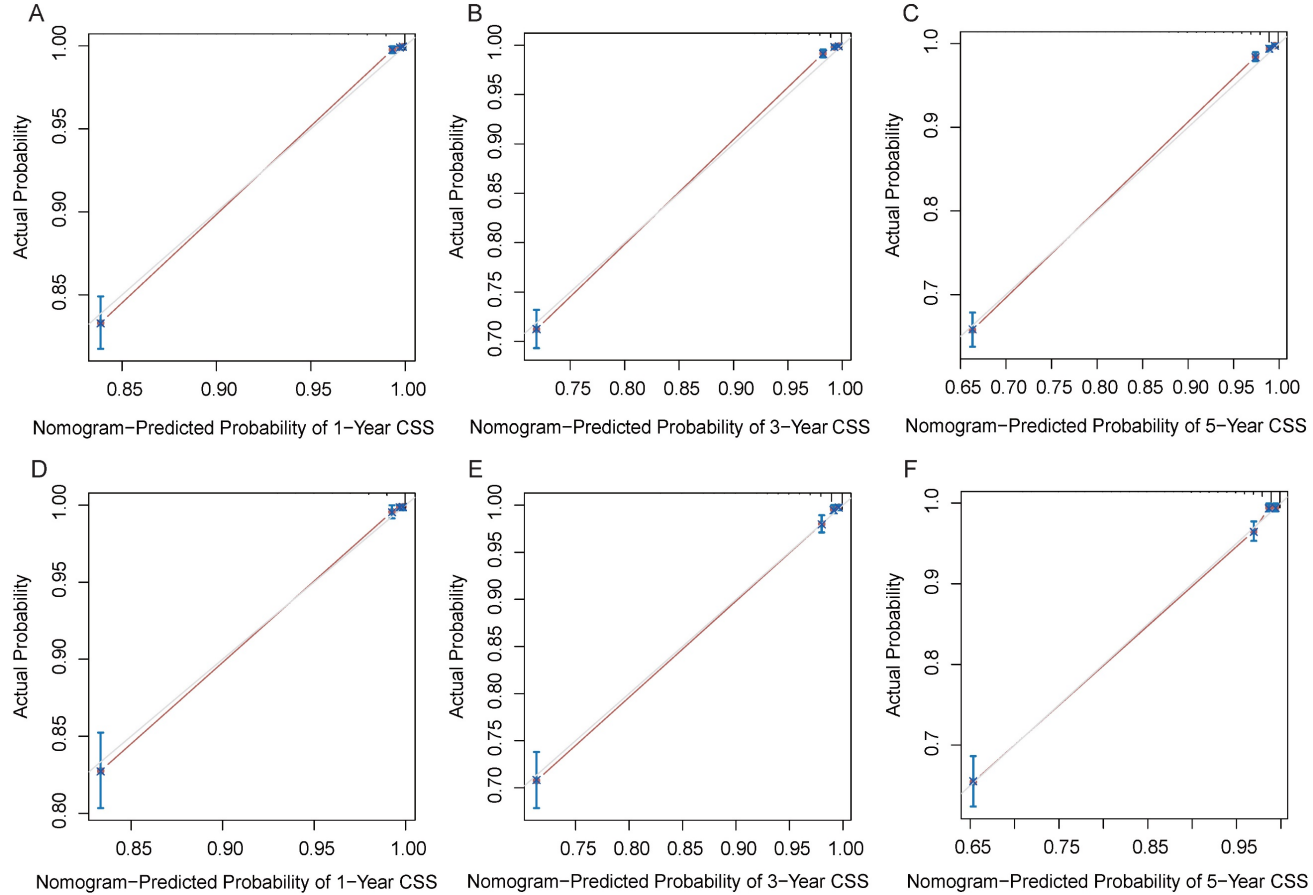
Calibration curves of the nomogram. (A) 1-year CSS in the training cohort. (B) 3-year CSS in the training cohort. (C) 5-year CSS in the training cohort. (D) 1-year CSS in the validation cohort. (E) 3-year CSS in the validation cohort. (F) 5-year CSS in the validation cohort.

In the training and validation cohorts, the C-index of the nomogram for OS were 0.834 and 0.828, respectively, indicating that the nomogram had excellent distinguishability. In the temporal and spatial external validation cohort, the C-index was 0.872 and 0.875, respectively. While the C-index of the nomogram for CSS in the training, validation, temporal and spatial external validation cohorts were 0.923, 0.828, 0.872 and 0.851, respectively. In the training cohort, the 1-year, 3-year and 5-year AUC of the nomogram for OS were 0.933, 0.912 and 0.884, respectively (Fig. 3A). In the validation cohort, the 1-year, 3-year and 5-year AUC of the nomogram for OS were 0.922, 0.898 and 0.872, respectively (Fig. 3B). In the temporal external validation cohort, the 1-year, 3-year and 5-year AUC of the nomogram for OS were 0.906, 0.899 and 0.876, respectively (Fig. 3C). The results indicated that the nomogram had high predictive ability. In addition, the AUC showed that the nomogram had high predictive ability for CSS (Fig. 3D-F). The 1-year, 3-year and 5-year calibration curves were also conducted to assess the difference in survival probability between the nomogram's prediction and the actual observation, and the results showed that the predicted curves approximatively overlapped with the diagonal line in the training cohort (Fig. 4A-C) and the validation cohort (Fig. 4D-F), suggesting the high prediction accuracy of the nomogram for OS. The 1-year, 3-year and 5-year calibration curves for CSS also showed the high prediction accuracy of the nomogram (Fig. 5A-F).

### Clinical application of the nomogram

**Figure 6:**
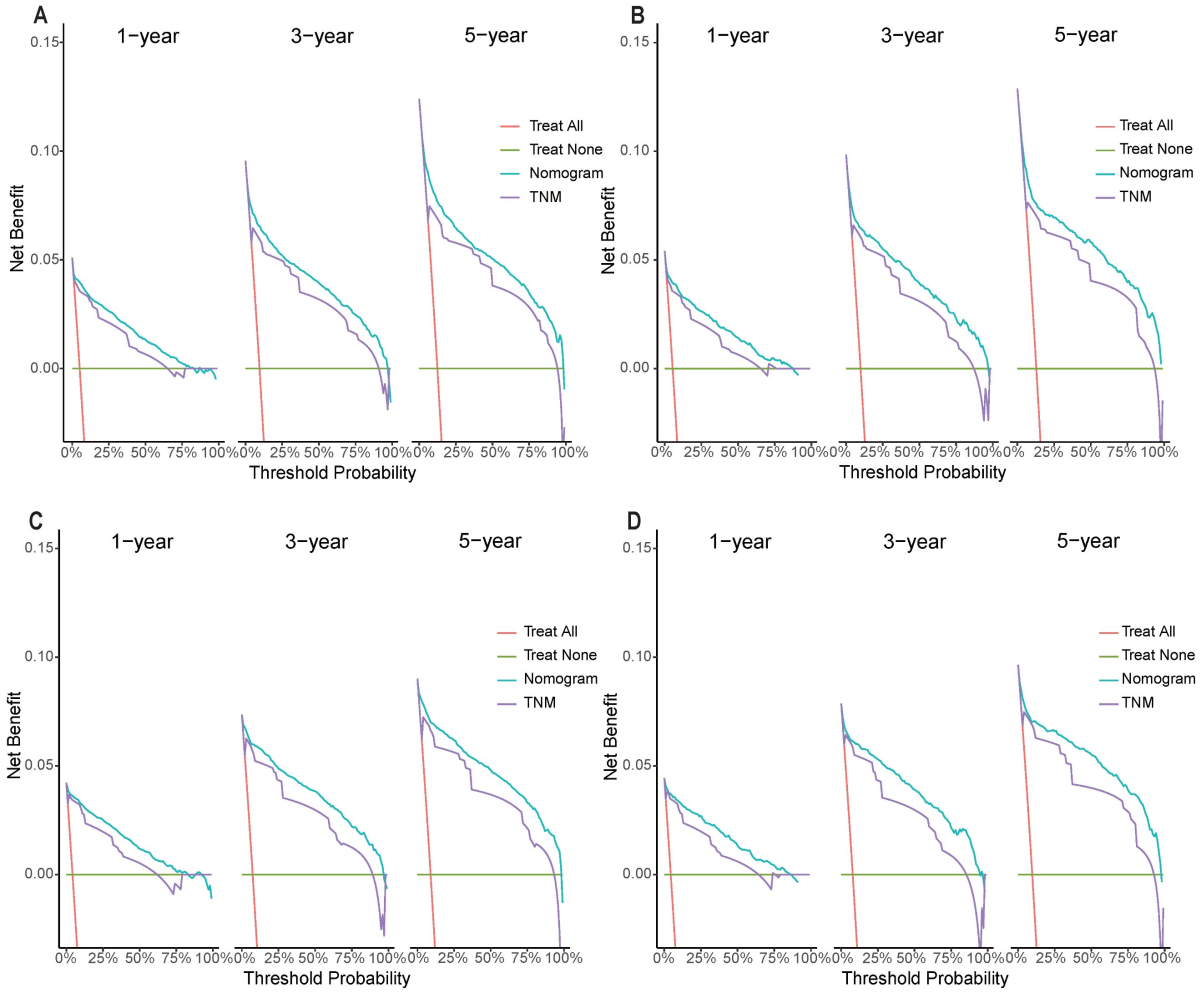
Decision curves of the nomogram predicting OS and CSS in the training cohort. (A) DCA curves of the nomogram predicting OS in the training cohort. (B) DCA curves of the nomogram predicting OS in validation cohort. (C) DCA curves of the nomogram predicting CSS in the training cohort. (D) DCA curves of the nomogram predicting CSS in validation cohort.

**Figure 7:**
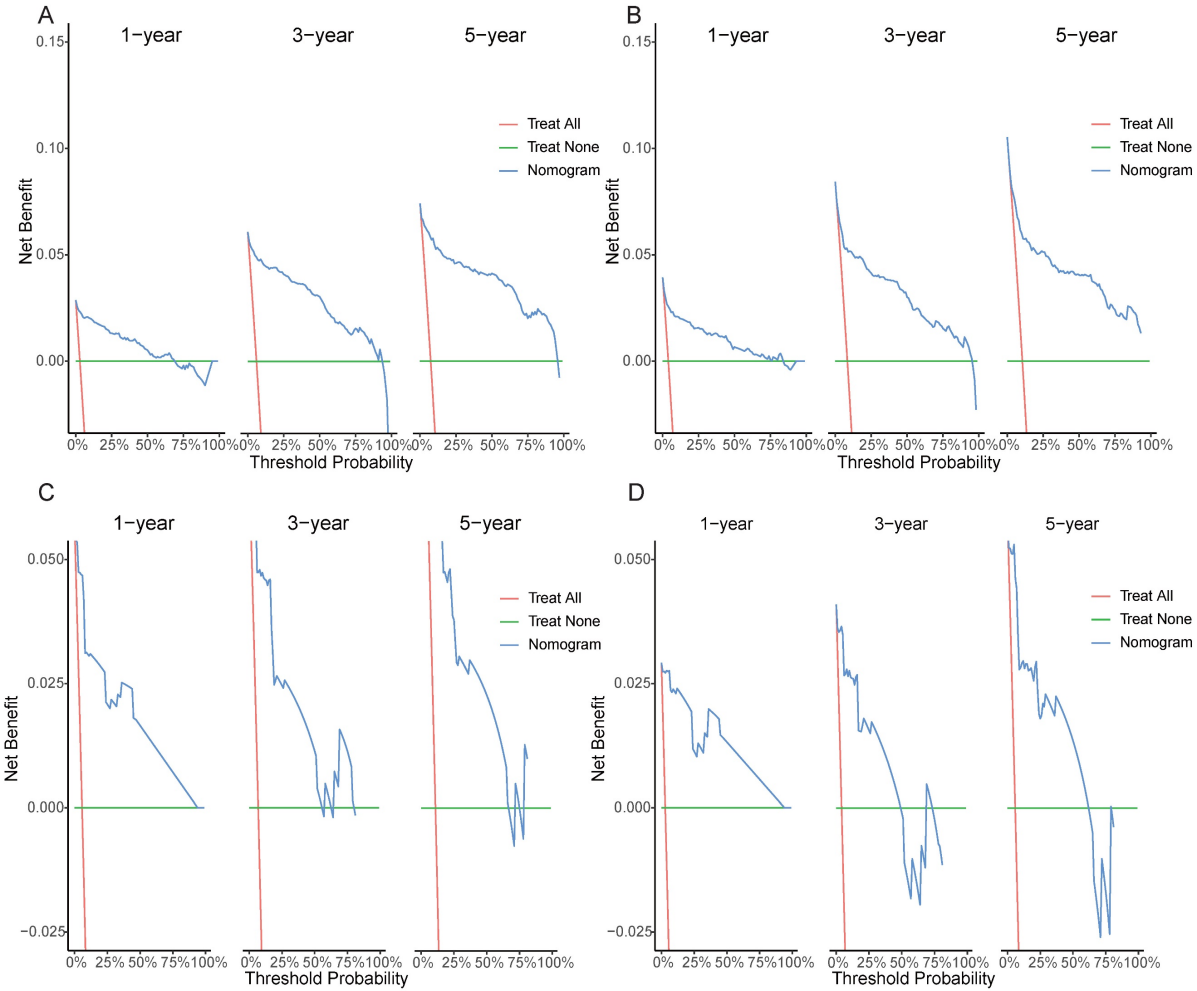
Decision curves of the nomogram predicting OS and CSS in both temporal and spatial external validation cohort. (A) DCA curves of the nomogram predicting OS in the temporal external validation cohort. (B) DCA curves of the nomogram predicting OS in the spatial external validation cohort. (C) DCA curves of the nomogram predicting CSS in the temporal external validation cohort. (D) DCA curves of the nomogram predicting CSS in the spatial external validation cohort.

**Figure 8:**
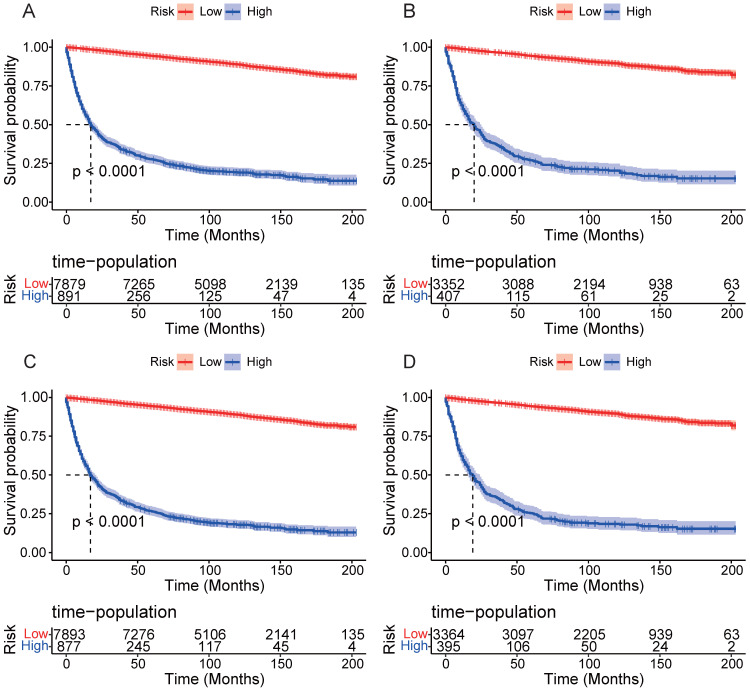
Kaplan-Meier curves of OS and CSS for patients in the low- and high-risk groups. (A) Kaplan-Meier curves of OS for patients in the low- and high-risk groups in the training cohort. (B) Kaplan-Meier curves of OS for patients in the low- and high-risk groups in the validation cohort. (C) Kaplan-Meier curves of CSS for patients in the low- and high-risk groups in the training cohort. (D) Kaplan-Meier curves of CSS for patients in the low- and high-risk groups in the validation cohort.

DCA results showed that applying the nomogram to guide clinical practice could provide EOKC patients with more net benefits than the TNM stage system in the training and validation cohorts, especially the long-term benefits at 3 and 5 years (Fig. 6A-D). The DCA of the temporal and spatial external validation cohort also suggested the high clinical value of the nomogram (Fig. 7A-D). To further optimize the clinical application of the nomogram, we developed a risk stratification system based on the total scores of patients in the nomogram. Patients were divided into a low-risk group (total score ≤ 280.1) and a high-risk group (total score > 280.1). In the training and validation cohorts, patients showed shorter OS in the high-risk group than in the low-risk group (Fig. 8A, B). The CSS of patients showed similar results (Fig. 8C, D).

## Discussion

With the development of medical imaging technology, an increasing number of young patients have been diagnosed with renal cancer. An epidemiological study shows that the incidence of RCC in young people is rapidly increasing, and the average annual growth rate is higher than that in the middle-aged and elderly 8. In addition, there are some differences in pathology, genetics and prognosis between young and old RCC patients 9. Accurate prediction of the prognosis of patients with EOKC can improve treatment strategies, survival outcomes and follow-up care.

At present, the research on the prognosis of EOKC is very limited, and only one study has been reported 18, in which the authors constructed a nomogram of EOKC and internally validated its performance. In this study, we included more clinicopathological factors, such as marital status, tumor size, and surgical methods, and performed the temporal and spatial external validation. The results showed that the proposed nomogram exhibited high prediction performance in both internal and external validations.

The two novel nomograms were constructed using a large number of clinical samples extracted from the SEER database to predict the prognosis of patients with EOKC. In clinical research, doctors and researchers often use the TNM staging system to evaluate patients' prognosis and make clinical decisions. However, compared to the traditional TNM staging system, recent studies have shown that the nomogram constructed based on clinicopathological data has higher accuracy in predicting patient survival 20, 21. Therefore, urologists can use the nomogram to evaluate the prognosis of EOKC patients and develop effective and individualized treatment strategies, thereby reducing the risk of death.

Multivariate cox regression analysis showed that age, sex, race, marital status, tumor grade, TNM stage, AJCC stage, tumor size, radiotherapy and surgical methods had significant effects on OS. These factors except for age and marital status were also independent prognostic factors for CSS in patients with EOKC. Age has been proven to be a key factor in the prognosis of various cancers 22-24. With increasing age, the patient's immune function and physical condition are declining, which may lead to tumor deterioration and an increased risk of death. This is also consistent with our previous research 25. Epidemiological studies show that the incidence and degree of malignancy of RCC are higher in males than in females 26, 27, which may be related to sex hormones. In addition, Ning et al. also found that the androgen-androgen receptor axis causes the exhaustion of CD8+ T-cells in the tumor microenvironment of male RCC patients 27, resulting in impaired anti-tumor function of these cells. All of these may lead to poor prognosis. Stafford et al. analyzed the relationship between demographic factors and death in patients with kidney cancer based on the California Cancer Registry and found that the survival rate of black patients is lower than that of whites and other races in the USA 28. Alam et al. found a similar result based on the National Cancer Database in the US 29. These results are also consistent with ours. In addition, this study showed that marital status was a significant predictor of OS in RCC patients. Married patients can receive more emotional comfort and financial support from their family partners, resulting in a better prognosis 30, 31. This is consistent with the previous research 15, 32.

Tumor grade is another important prognostic factor for patients with EOKC. Our results are consistent with previous reports 33-36. In general, a high tumor grade indicates a low degree of differentiation but a high degree of malignancy, which may result in a poor prognosis. It has become a consensus that the TNM stage can affect the prognosis of patients with many kinds of tumors 37. In this study, the nomogram showed that the AJCC stage made a greater contribution to the prognosis of patients compared with T stage, N stage and M stage. This is because the AJCC stage is a combination of T, N and M stages to assess the prognosis of patients with EOKC, indicating that this staging system had a more clinical value. Our results also showed that radiotherapy could be used to predict the prognosis of patients, possibly due to the fact that patients undergoing radiotherapy often have metastasis, which results in a poor prognosis. At the same time, radiotherapy has been considered a double-edged sword. Long-term radiotherapy can cause damage to the immune system and physiological function, which may lead to severe disability and mortality 38, 39. In this study, we found that patients undergoing PN had the longest OS, followed by those who underwent local tumor resection, RN and non-surgery treatment. This aligns with previous research findings related to RCC 15, 40. Local RCC can usually be removed by surgery and have a good prognosis. Among them, patients treated with PN had a better prognosis than those treated with RN. Generally, patients undergoing RN typically present tumors situated in intricate locations or infiltrating adjacent organs. In such cases, patients are more likely to develop distant metastasis even after surgery 41. The prognosis is usually poor for patients who have not undergone surgery, since they are often in advanced stages or have metastasis.

The nomogram constructed in this study included demographic and clinicopathological factors, which are easy to collect in clinical practice. Across the four cohorts, this nomogram exhibited higher C-index and AUC values, suggesting that it had high accuracy and discrimination. In the training and internal validation cohorts, the calibration curves also showed a promising prediction performance. However, despite high accuracy and discriminative ability, their clinical applicability remains uncertain. Therefore, we conducted DCA and confirmed that the clinical net benefit of the nomogram outweighed that of the TNM staging system, particularly long-term benefits at 3 and 5 years. In addition, we utilized the risk scores derived from the nomogram to stratify the patients into different risk groups, and the results showed that the prognosis of patients in the high-risk group was significantly lower than that in the low-risk group. The results suggest that clinicians can identify high-risk patients according to the risk scores and implement appropriate therapeutic strategies to improve the prognosis of patients. Full evaluation should be made for high-risk patients, appropriate targeted therapy and immunotherapy should be used, and follow-up intervals should be shortened.

This study has several limitations. First, as a retrospective study, it faces challenges related to selection bias. Therefore, it is needed to conduct a large-scale prospective clinical study to validate the accuracy of the nomogram in the future. Second, the temporal external validation cohort for this study includes data from 2015, which is relatively early. However, incorporating data between 2013 and 2021 from our hospital for spatial external validation still demonstrates robust predictive performance. Additionally, the SEER database lacks information on comorbidities, such as complications, hypertension, BMI, etc. Nevertheless, the nomogram took some crucial determinants, such as tumor stages and grades, into account, and it still had great potential to be used in clinical practices.

## Conclusion

In this study, we constructed a novel nomogram for predicting the prognosis of patients with EOKC based on the large-scale clinical data derived from the SEER database. This nomogram exhibited higher prediction performance. Clinicians can utilize this tool to assess the prognostic risk of patients and formulate effective intervention and follow-up strategies. In the future, more prospective clinical studies are essential to validate this nomogram and improve its clinical applicability.

## Figures and Tables

**Table 1:** Clinicopathological characteristics of patients with EOKC

	Training cohort	Validation cohort	Temporal external validation	*P*-value
	(N=8770)	(N=3759)	(N=1498)	
Age (years)	39.5 (6.15)	39.5 (6.03)	39.6 (5.76)	0.713/0.827
Sex				0.823/0.117
Female	3355 (38.3%)	1446 (38.5%)	605 (40.4%)	
Male	5415 (61.7%)	2313 (61.5%)	893 (59.6%)	
Race				0.943/0.168
Black	1102 (12.6%)	467 (12.4%)	195 (13.0%)	
other	696 (7.9%)	304 (8.1%)	139 (9.3%)	
White	6972 (79.5%)	2988 (79.5%)	1164 (77.7%)	
Marital status				0.764/0.433
Married	4972 (56.7%)	2142 (57.0%)	833 (55.6%)	
other	3798 (43.3%)	1617 (43.0%)	665 (44.4%)	
Histologic subtypes				0.830/0.004
Clear cell	5070 (57.8%)	2171 (57.8%)	890 (59.4%)	
RCC	1709 (19.5%)	713 (19.0%)	292 (19.5%)	
Papillary	821 (9.4%)	346 (9.2%)	104 (6.9%)	
Chromophobe	694 (7.9%)	313 (8.3%)	144 (9.6%)	
other	476 (5.4%)	216 (5.7%)	68 (4.5%)	
Grade				0.035/0.528
I/II	5146 (58.7%)	2210 (58.8%)	866 (57.8%)	
III/IV	2113 (24.1%)	963 (25.6%)	381 (25.4%)	
Unknown	1511 (17.2%)	586 (15.6%)	251 (16.8%)	
T stage				0.881/0.443
T1/T2	7576 (86.4%)	3251 (86.5%)	1283 (85.6%)	
T3/T4	1194 (13.6%)	508 (13.5%)	215 (14.4%)	
N stage				0.453/0.943
N0	8370 (95.4%)	3577 (95.2%)	1431 (95.5%)	
N1	223 (2.5%)	93 (2.5%)	36 (2.4%)	
N2	177 (2.0%)	89 (2.4%)	31 (2.1%)	
M stage				0.881/0.546
M0	8207 (93.6%)	3515 (93.5%)	1408 (94.0%)	
M1	563 (6.4%)	244 (6.5%)	90 (6.0%)	
AJCC Stage				0.810/0.625
I	6348 (72.4%)	2702 (71.9%)	1087 (72.6%)	
II	962 (11.0%)	432 (11.5%)	159 (10.6%)	
III	813 (9.3%)	341 (9.1%)	151 (10.1%)	
IV	647 (7.4%)	284 (7.6%)	101 (6.7%)	
Tumor size (cm)	5.05 (4.08)	5.03 (3.93)	4.91 (3.60)	0.761/0.147
Radiotherapy				0.327/0.117
No/Unknown	8567 (97.7%)	3661 (97.4%)	1473 (98.3%)	
Yes	203 (2.3%)	98 (2.6%)	25 (1.7%)	
Chemotherapy				0.175/0.8
No/Unknown	8327 (94.9%)	3547 (94.4%)	1420 (94.8%)	
Yes	443 (5.1%)	212 (5.6%)	78 (5.2%)	
Surgery				0.449/0.013
No	384 (4.4%)	151 (4.0%)	77 (5.1%)	
Local excision	189 (2.2%)	89 (2.4%)	46 (3.1%)	
Partial Nephrectomy	3370 (38.4%)	1407 (37.4%)	605 (40.4%)	
Radical Nephrectomy	4827 (55.0%)	2112 (56.2%)	770 (51.4%)	
Survival months	110(52.0)	111(53.1)	57.2 (18.9)	0.693/<0.001
Overall survival				0.778/0.191
Dead	1659 (18.9%)	703 (18.7%)	262 (17.5%)	
Alive	7111 (81.1%)	3056 (81.3%)	1236 (82.5%)	
Cancer-specific survival				0.838/0.228
Dead	1013 (11.6%)	439 (11.7%)	157 (10.5%)	
Alive	7757 (88.4%)	3320 (88.3%)	1341 (89.5%)	

**Table 2:** Univariable and multivariable Cox regression analysis of OS for EOKC patients in the training cohort

	Univariate			Multivariable		
	HR	95%CI	*P*	HR	95%CI	*P*
Age (years)	1.025	1.016~1.034	<0.001	1.022	1.014~1.031	<0.001
Sex						
Female	Reference					
Male	1.501	1.351~1.668	<0.001	1.269	1.140~1.412	<0.001
Race						
Black	Reference					
White	0.526	0.465~0.593	<0.001	0.649	0.570~0.739	<0.001
Other	0.559	0.455~0.687	<0.001	0.681	0.551~0.841	<0.001
Marital status						
Married	Reference					
Other	1.527	1.387~1.682	<0.001	1.405	1.272~1.553	<0.001
Histologic subtypes						
Clear cell	Reference					
Papillary	1.344	1.140~1.584	<0.001	1.078	0.907~1.282	0.395
Chromophobe	0.328	0.237~0.455	<0.001	0.339	0.243~0.472	<0.001
RCC	1.781	1.587~2.000	<0.001	1.181	1.042~1.338	0.009
Other	3.572	3.063~4.166	<0.001	1.715	1.455~2.021	<0.001
Grade						
I/II	Reference					
III/IV	4.018	3.591~4.495	<0.001	1.854	1.638~2.100	<0.001
Unknown	3.122	2.743~3.554	<0.001	1.452	1.246~1.693	<0.001
T stage						
T1/T2	Reference					
T3/T4	5.871	5.315~6.485	<0.001	1.441	1.217~1.707	<0.001
N stage						
N0	Reference					
N1	13.910	11.910~16.250	<0.001	1.977	1.651~2.366	<0.001
N2	18.620	15.710~22.070	<0.001	1.721	1.388~2.133	<0.001
M stage						
M0						
M1	23.350	20.880~26.120	<0.001	2.046	1.504~2.782	<0.001
AJCC stage						
I	Reference		<0.001			
II	1.677	1.411~1.993	<0.001	1.325	1.097~1.602	0.004
III	4.058	3.527~4.668	<0.001	1.899	1.535~2.349	<0.001
IV	28.823	25.620~32.426	<0.001	4.102	2.954~5.697	<0.001
Tumor size (cm)	1.055	1.051~1.059	<0.001	1.009	1.000~1.018	0.043
Radiotherapy						
No/unknown	Reference					
Yes	16.480	14.110~19.240	<0.001	1.217	1.016~1.457	0.033
Chemotherapy						
No/unknown	Reference					
Yes	14.090	12.510~15.870	<0.001	1.114	0.942~1.318	0.209
Surgery						
No	Reference					
Local excision	0.078	0.051~0.121	<0.001	0.286	0.183~0.449	<0.001
Partial Nephrectomy	0.045	0.038~0.054	<0.001	0.189	0.152~0.236	<0.001
Radical Nephrectomy	0.158	0.138~0.181	<0.001	0.325	0.273~0.388	<0.001

**Table 3:** Univariable and multivariable Cox regression analysis of CSS for EOKC patients in the training cohort

	Univariate			Multivariable		
	HR	95%CI	*P*	HR	95%CI	*P*
Age (years)	1.010	1.000~1.020	0.032	1.009	0.999~1.018	0.082
Sex						
Female	Reference					
Male	1.590	1.390~1.820	<0.001	1.316	1.144~1.512	<0.001
Race						
Black	Reference					
White	0.545	0.466~0.638	<0.001	0.690	0.583~0.816	<0.001
Other	0.637	0.493~0.822	<0.001	0.755	0.580~0.983	0.037
Marital status						
Married	Reference					
Other	1.290	1.140~1.460	<0.001	1.099	0.965~1.251	0.156
Histologic subtype						
Clear cell	Reference					
Papillary	1.189	0.943~1.500	0.140	0.994	0.779~1.269	0.964
Chromophobe	0.284	0.177~0.455	<0.001	0.279	0.173~0.450	<0.001
RCC	2.109	1.820~2.444	<0.001	1.296	1.104~1.521	0.002
Other	5.354	4.491~6.382	<0.001	1.937	1.600~2.346	<0.001
Grade						
I/II	Reference					
III/IV	8.250	7.010~9.710	<0.001	2.367	1.982~2.827	<0.001
Unknown	5.170	4.290~6.230	<0.001	1.565	1.256~1.949	<0.001
T stage						
T1/T2	Reference					
T3/T4	10.600	9.330~12.000	<0.001	1.285	1.075~1.534	0.006
N stage						
N0	Reference					
N1	22.400	18.900~26.500	<0.001	1.956	1.618~2.363	<0.001
N2	29.000	24.100~34.800	<0.001	1.648	1.316~2.064	<0.001
M stage						
M0						
M1	40.400	35.400~46.100	<0.001	1.948	1.408~2.694	<0.001
AJCC stage						
I	Reference		<0.001			
II	4.610	3.610~5.900	<0.001	3.044	2.339~3.961	<0.001
III	12.820	10.450~15.700	<0.001	5.377	4.112~7.030	<0.001
IV	98.880	82.340~118.800	<0.001	12.197	8.377~17.759	<0.001
Tumor size (cm)	1.060	1.060~1.070	<0.001	1.014	1.005~1.023	0.002
Radiotherapy						
No/unknown	Reference					
Yes	23.200	19.600~27.300	<0.001	1.263	1.048~1.521	0.014
Chemotherapy						
No/unknown	Reference					
Yes	22.900	20.000~26.200	<0.001	1.137	0.953~1.357	0.154
Surgery						
No	Reference					
Local excision	0.015	0.005~0.047	<0.001	0.123	0.039~0.388	<0.001
Partial Nephrectomy	0.015	0.011~0.020	<0.001	0.130	0.092~0.185	<0.001
Radical Nephrectomy	0.150	0.128~0.175	<0.001	0.342	0.278~0.421	<0.001

## Abbreviations

RCC: renal cell carcinoma
EOKC: early-onset kidney cancer
SEER: Surveillance Epidemiology and End Results
ccRCC: clear cell renal cell carcinoma
pRCC: papillary renal cell carcinoma
chRCC: chromophobe renal cell carcinoma
ROC: receiver operating characteristic
PN: partial nephrectomy
RN: radical nephrectomy
HR: hazard ratio
CI: confidence interval
OS: overall survival
CSS: cancer-specific survival
AUC: area under the ROC curve
C-index: Concordance Index
DCA: Decision curve analysis
